# The Role of Sperm Proteins IZUMO1 and TMEM95 in Mammalian Fertilization: A Systematic Review

**DOI:** 10.3390/ijms23073929

**Published:** 2022-04-01

**Authors:** Miranda Hernández-Falcó, Paula Sáez-Espinosa, Andrea López-Botella, Jon Aizpurua, María José Gómez-Torres

**Affiliations:** 1 Departamento de Biotecnología, Facultad de Ciencias, Universidad de Alicante, 03690 Alicante, Spain; mhf7@alu.ua.es (M.H.-F.); paula.saez@ua.es (P.S.-E.); andrea.lopez@ua.es (A.L.-B.); 2 IVF Spain, Reproductive Medicine, 03540 Alicante, Spain; j.aizpurua@ivf-spain.com; 3 Cátedra Human Fertility, Facultad de Ciencias, Universidad de Alicante, 03690 Alicante, Spain

**Keywords:** fertilization, gamete fusion, IZUMO1, mammals, reproduction, spermatozoa, TMEM95 (transmembrane protein 95)

## Abstract

Gamete membrane fusion is a critical cellular event in sexual reproduction. In addition, the generation of knockout models has provided a powerful tool for testing the functional relevance of proteins thought to be involved in mammalian fertilization, suggesting IZUMO1 and TMEM95 (transmembrane protein 95) as essential proteins. However, the molecular mechanisms underlying the process remain largely unknown. Therefore, the aim of this study was to summarize the current knowledge about IZUMO1 and TMEM95 during mammalian fertilization. Hence, three distinct databases were consulted—PubMed, Scopus and Web of Science—using single keywords. As a result, a total of 429 articles were identified. Based on both inclusion and exclusion criteria, the final number of articles included in this study was 103. The results showed that IZUMO1 is mostly studied in rodents whereas TMEM95 is studied primarily in bovines. Despite the research, the topological localization of IZUMO1 remains controversial. IZUMO1 may be involved in organizing or stabilizing a multiprotein complex essential for the membrane fusion in which TMEM95 could act as a fusogen due to its possible interaction with IZUMO1. Overall, the expression of these two proteins is not sufficient for sperm–oocyte fusion; therefore, other molecules must be involved in the membrane fusion process.

## 1. Introduction

The fertilization process is a vital step in sexual reproduction that entails a series of synchronized events to produce a zygote that is genetically unique. After ejaculation, millions of sperm are deposited in the female reproductive tract. However, only a few of these cells will reach the ampulla of the oviduct and meet the oocyte. Sperm acquire the ability to fertilize oocytes during this transit through a physiological and molecular changes known as capacitation [1]. As a consequence of capacitation, spermatozoa exhibit three fertility-related modifications: changes in sperm motility patterns; regulation of signal transduction pathways that allow them to respond to chemoattractants; and the ability of sperm to undergo the AR (acrosome reaction) [2,3,4]. Upon ovulation, only one spermatozoon successfully fuses with the oocyte.

It is known that fertilization occurs in four successive steps. First, the sperm must undergo the AR to release the enzymes and ligands necessary for the fertilization [5]. Sperm-reacted can fuse with the oocyte plasma membrane through a remnant of the sperm plasma membrane lying over the equatorial segment (EQ) [6]. As a second step, sperm need to penetrate the zona pellucida (ZP) in order to reach the perivitelline space, which is an extensive gap between the oocyte and the ZP [7]. Thirdly, the acrosome-reacted sperm must adhere to the oolemma; this step is highly specific to mammalians [8,9]. 

Cellular adhesion is determined by known or putative molecular interactions between sperm proteins and the oolemma. Following gamete adhesion, the formation of fusion pores allows cytoplasmic continuity and gamete fusion [9]. Fusogen proteins are responsible for facilitating membrane fusion during cell interactions [6]. The generation of knockout models has provided a powerful tool for testing the functional relevance of proteins proposed to have a role in mammalian fertilization contributing to the construction of a new scheme of fertilization mechanism. 

In this way, through using loss-of-function experiments in transgenic or mutant mice, researchers have proven that two sperm proteins are essential for the sperm to adhere to the oolemma during fertilization: IZUMO1 [10,11] and SPACA6 (sperm acrosome membrane-associated protein 6) [12]. Similarly, oocyte proteins, such as the tetraspanins CD9 [13] and CD81 [14] and JUNO [15], are also required. Females lacking these proteins have a marked phenotype; despite exhibiting a normal behaviour and being able to produce oocytes that are normal in appearance, their fertilisation fails at the final adhesion and fusion steps.

Specifically, IZUMO1 protein is a testis-specific cell-surface protein belonging to the class of immunoglobulin type-I cell superfamily, characterized by a cytoplasmic C-terminal tail, a transmembrane region and a conserved ‘Izumo domain’, which is linked to an extracellular immunoglobulin-like (Ig-like) C2-type domain. It has been demonstrated that each of these domains plays a critical role in gamete adhesion [10,16,17]. After AR in mammalians, IZUMO1 is localized in the EQ [18] to mediate gamete adhesion with the oocyte plasma membrane. The absence of IZUMO1 leads to the impairment of gamete adhesion and to an accumulation of sperm in the perivitelline space [10]. However, despite being a protein necessary to carry out membrane fusion, it lacks a fusogenic peptide or SNARE-like structure, and thus IZUMO1 could be one of the components that form the fusogenic machinery in spermatozoa, acting at the level of organization or stabilization of a multiprotein complex [19].

Recently, three new sperm proteins that are essential for mammalian fertilization have been identified through the use of CRISPR technologies: TMEM95 (transmembrane protein 95), SOF1 (Sperm-egg fusion protein LLCFC1) [20] and FIMP (Fertilization-influencing membrane protein) [21]; these are small proteins that are expressed highly in the testis. Male mice lacking any one of these proteins phenocopied IZUMO1-deficient males; they produced sperm with normal morphology and motility, and their passage of the ZP and binding to oocytes were comparable with that of wild-type sperm. Nevertheless, this final step was unsuccessful, and the sperm was unable to fuse with the oocyte. These articles propose that one of these three proteins functions as a fusogen during fertilization.

TMEM95 is another essential protein for performing fertilization and is highly conserved in mammalians; therefore, its alterations could induce male subfertility [22]. After AR, TMEM95 localizes in the EQ as IZUMO1. However, in bovines, other localization patterns have been observed, including in the neck, thereby, indicating an additional role during the first cell divisions of the embryo [23] and its disappearance after AR implying a different function from IZUMO1 [24]. Due to the localization of IZUMO1 and TMEM95 in the EQ after AR and their co-immunoprecipitation, this suggests a possible interaction between them. Therefore, TMEM95 could act as a fusogen or mask the real fusogen.

Gamete adhesion and fusion have been extensively studied in recent decades. Researchers have monitored in vitro fertilization (IVF) studies to see the consequences of defective adhesion and fusion of mammalian gametes. Despite their importance in sexual reproduction, the molecular mechanisms involved in gamete membrane fusion remain largely unknown. Here, we analysed bibliometrically and bibliographically the publications on TMEM95 and IZUMO1, two proteins highly conserved among mammalian species that could interact with each other, to summarize the information and aid in the construction of the mammalian fertilization mechanism, in order to elucidate idiopathic male infertility.

## 2. Materials and Methods 

### 2.1. Search Strategy and Information Processing

The methodology followed was based on previous studies performed by our research group [25,26]. First, generic searched were performed using the “Google scholar” portal (https://scholar.google.es/, accessed on 4 November 2021) to obtain keywords that would subsequently be used in a specialized search in scientific databases. Due to the scarcity of publications, the final keywords were “TMEM95” and “IZUMO1”. 

A full search was performed in online databases related to the issue under study to achieve an accurate bibliometric and bibliographic analysis and to be aware of the bibliographic load indexed in each one of the online databases. The selected databases were PubMed, Web of Science (WOS) and Scopus. In the WOS database, the “Search all database” option and the field tag “Topic” were selected during this search. In Scopus database the field tag “Article title, Abstract, Keywords” was selected during this search.

### 2.2. Selection of Relevant Studies and Data Analysis

The initial search was performed, as described above, using the keywords “TMEM95”and “IZUMO1” in each one of the databases. An attached database was created in RefWorks to include the articles obtained from each search result.

**Figure 1:**
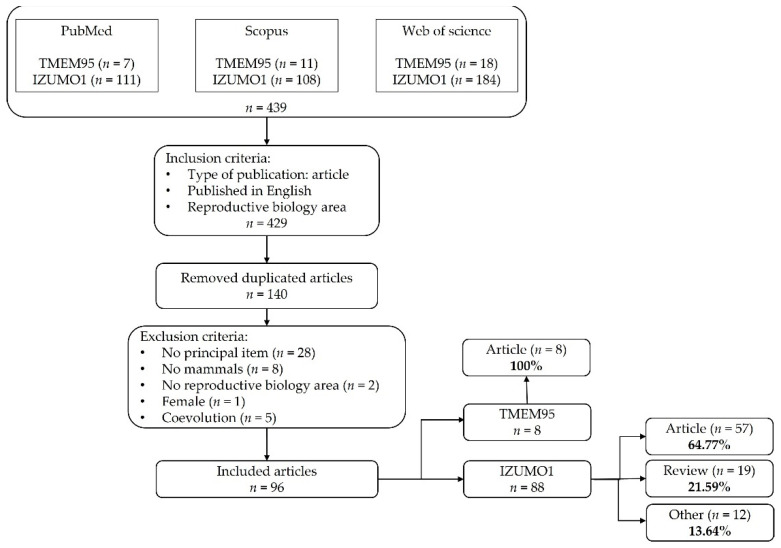
Flowchart summarizing the selection process of the articles included in the present review regarding IZUMO1 and TMEM95. Adapted from PRISMA Group [27].

After that, we incorporated two inclusion criteria that helped to filter the results and served to select the documents: (i) articles from primary sources and indexed journals and (ii) studies published in English. WOS was the only database that allowed selection of the research area (Reproductive Biology) as an inclusion criterion. Then, the duplicated articles were removed from the RefWorks database. In addition, to build the final database we applied the following accorded exclusion criteria: (i) studies not related to males; (ii) non-mammalian studies; (iii) studies not related to reproductive biology; (iv) studies without TMEM95 or IZUMO1 as the main topic; and (v) coevolution studies. The final number of articles included in this review was 97 (view Figure 1). Finally, due to the redundancy of information, only TMEM95 and IZUMO1 original articles were used and cited in this systematical review.

## 3. Results and Discussion

### 3.1. Compilation of Relevant Bibliographic Sources

Initially, a total of 439 articles were found after using the keywords in PubMed, Scopus and WOS, which became 429 after applying our inclusion criteria. Then, 289 duplicate articles were removed. According to the exclusion criteria, a total amount of 44 articles were discarded. Finally, a total of 96 articles were left—21.87% of the total articles initially found—forming the database to be ultimately analysed. The analysis of these article revealed that eight articles studied TMEM95; while 88 had IZUMO1 as main topic of which 57 were articles (64.77%), 19 were reviews (21.59%), and 12 were other types, such as chapters, letters and conference materials (13.64%) (Figure 1). Our Supplementary Materials are available with information for every type of article reviewed (Tables S1–S4).

### 3.2. Bibliometric Analysis

**Figure 2:**
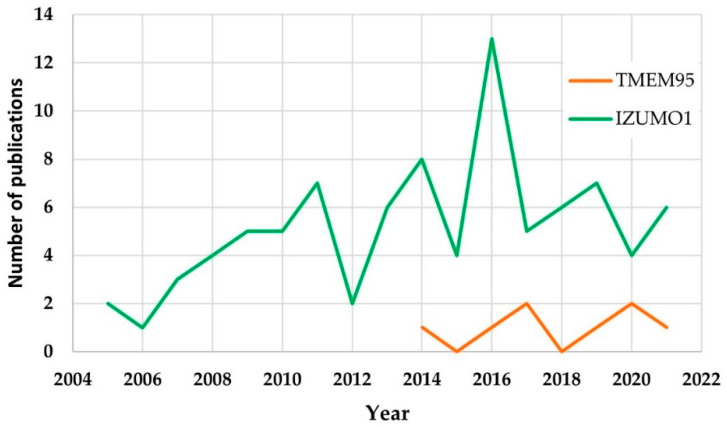
The number of publications as a function of the year (March 2005–May 2021), including all the articles from the database created after the application of inclusion and exclusion criteria for IZUMO1 (green) and TMEM95 (orange).

The keyword search showed that the number of publications relating TMEM95 were lower than those related to IZUMO1. This could be explained by the description of the protein TMEM95 in 2014 [23], while the first publications on IZUMO1 began in 2005 [10]. The analysis of the 88 articles about IZUMO1 revealed that the highest value in the number of publications was reached in 2016 with 13 publications (Figure 2). Furthermore, an increase in the number of publications after 2012 is noticed. Specifically, 50.6% of the articles had been published since 2015. These findings could be explained by an increase in the interest in idiopathic male infertility and/or the advances in the clarification of the molecular mechanism of fertilization [28].

The molecular mechanism of fertilization has been studied in diverse countries. However, when we focused on the authorship of the documents, the studies were conducted in countries with high idiopathic infertility in men, such as China (25%) [29] and Japan (42.1%) [30].

### 3.3. Bibliographical Analysis

**Figure 3:**
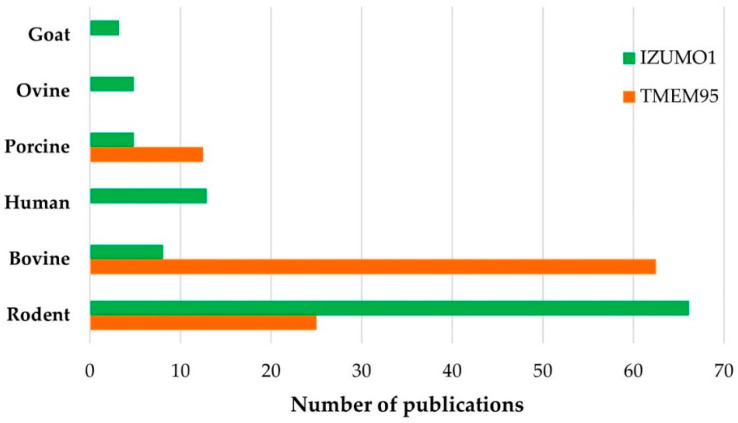
The number of publications according to the species studied in articles for IZUMO1 (green) and TMEM95 (orange).

Studies on the proteins involved in fertilization have been performed in a variety of species (Figure 3). TMEM95 research has focused on domestic animals, such as bovines [31] and boars [32], due to the great interest generated by their breeding. Regarding research on IZUMO1, because of its positive correlation with fertility, has focused on humans [33] and mouse [34] models. 

### 3.4. Analysis of TMEM95

The results showed that eight articles studied TMEM95 (Table 1). The main objectives of these articles were to molecularly characterize TMEM95 [20,22,31,35] or to determine the cause or effect of a certain mutation, including nonsense mutations [23,24], synonymous mutations [32] and insertion/deletion mutations [36].

TMEM95 encodes a highly conserved single pass type I transmembrane protein consisting of 176 amino acids. The presence of a Pfam:IZUMO domain—database of protein families and domains—and a leucine rich repeat C-terminal domain (LRRCT) has been predicted, the latter being involved in a multitude of biological functions, such as signal transduction and cell adhesion [37]. Its expression differs between species, being exclusive to the testis in rodents, while in cattle, it is also expressed in the brain. In addition, two splicing variants have been found in bovines [31]. In mature mammalian sperm, this protein relocalizes from the outer acrosomal membrane to the EQ after undergoing AR. It has also been localized to the neck of bovine spermatozoa, indicating an additional role of TMEM95 during early embryonic cell divisions [23]. This hypothesis was confirmed after observing a lower cleavage rate in embryos obtained from spermatozoa with mutated TMEM95 [24].

The protein sequence of TMEM95 is highly conserved among mammalian species, such as rodents, boars, bovines and primates, including humans [20]. Therefore, genetic variants altering TMEM95 are likely to induce male subfertility. In humans, numerous polymorphic sites have been identified, including several potential loss-of-function variants [23]. In other species (bovine and rodents), ablation or mutation of TMEM95 prevents penetration and fusion between the sperm and oocyte membranes, leading to sperm accumulation in the perivitelline space. This blockade of fusion does not generate structural defects in spermatozoa; therefore, it is suggested that TMEM95 may not play an important architectural role. TMEM95 deficiency is not detectable in a routine seminogram as it does not affect sperm motility or morphology.

Due to the normality of the seminogram, it might be advisable to develop functional assays, e.g., for the integrity of sperm-surface proteins or for effective prospective monitoring of male fertility [22,23,24].

Assays performed in HEK293T cells, noted by their adherent growth, suggest that TMEM95 requires one or more partners as, by itself, it does not promote cell fusion after IZUMO1 and JUNO binding [22]. Coimmunoprecipitation analyses suggest interactions of TMEM95, SPACA6, SOF1 and FIMP with IZUMO1 [20]. However, the nature of this cooperation is unknown as ablation of any of these genes does not affect the amount or localization of IZUMO1 [20,22,38]. It should be noted that the expression of these five proteins is not sufficient for sperm–oocyte fusion. 

On the other hand, no interactions between TMEM95 and IZUMO1 or JUNO have been detected [22]. Therefore, it is suggested that TMEM95, SOF1 and SPACA6 could directly or indirectly regulate membrane fusion through an IZUMO1-independent pathway or act as fusion mediators downstream of IZUMO1 and JUNO interaction. Membrane fusion is a highly coordinated and dynamic process, involving tight timing and proper interactions between molecules to allow the sperm head to internalize into the ooplasm [20]. 

Interestingly, TMEM95 disappears in bovine spermatozoa that have undergone the AR [24] implying a different function than IZUMO1. It is possible that TMEM95 does not act as a fusogenic protein that directly mediates membrane fusion between spermatozoa and oocytes. Rather, it could mask the actual fusogens that mediate fusion prior to AR; the release of TMEM95 could facilitate exposure of the functional domain of the fusogenic protein to the cell surface, which is necessary for fusion to occur. Alternatively, it is possible that sperm and oocyte membrane fusion is a bilateral fusion process, in which the fusogen must be present on both membranes; during acrosomal exocytosis, TMEM95 may be released and transferred to the oocyte plasma membrane, thus, ensuring that fusion occurs bilaterally [20].

### 3.5. Analysis of IZUMO1

#### 3.5.1. Characterization and Localization of IZUMO1

The characterization and localization of IZUMO1 has been studied in 21 articles (Table 2). In this case, a wide variety of species were analysed, including rodents, bovines, boars and humans. The samples were obtained from the testis, epididymis, fresh ejaculates and other tissues. IZUMO1 characterization was performed using different techniques, such as genotyping, cloning, mutation, deletion, expression and bioinformatic analysis.

IZUMO1 encodes a single-pass type I transmembrane protein consisting of 350 amino acids with a 21 amino acid extracellular N-terminal signal peptide, a 20 amino acid transmembrane domain (amino acid position 293 to 313) and a 36 amino acid intracellular C-terminal domain (human Uniprot IZUMO1) containing several potential phosphorylation sites [39]. It belongs to the immunoglobulin superfamily with an IZUMO domain and an extracellular immunoglobulin domain [40]. The IZUMO domain includes a receptor-binding platform IZUMO1 to JUNO, which is a glycosylphosphatidylinositol (GPI)-anchored oolemma protein [15,16,17]. An isoform of IZUMO1 (IZUMO1_v2) encoded by a different exon (exon 1b) of the *Izumo1* through alternative splicing has been observed in mice. This isoform exhibits functional properties identical to the original protein [41].

The IZUMO family consists of four proteins: IZUMO1, IZUMO2, IZUMO3 and IZUMO4. All of them share a region of residues of about 150 amino acids of high homology between them called IZUMO domain, located between the signal peptide and the immunoglobulin domain. Experiments with native and recombinant IZUMO proteins suggest that the IZUMO domain is involved in homodimer formation. These proteins, originally discovered in the mouse, have homologues in several mammalian species, thus it is a conserved family [39]. Western blotting has detected IZUMO1 as a 56.4 kDa protein in mice, whereas in human weights 37.2 kDa [10].

All members of the IZUMO family show a pattern of eight conserved cysteines and a similar predicted secondary structure consisting of four α-helices between cysteine motifs. Notably, IZUMO4 lacks a transmembrane domain [39]. IZUMO1, a stable monomeric protein with extensive mixed α-β secondary structural features, was characterized in 2016. The overall structure consists of two domains: a four-helical N-terminal rod-shaped bundle of four helices (4HB; residues 22–134) and an immunoglobulin-like domain (Ig-like; residues 167–254). Two antiparallel β-strands (β1 and β2) function as a hinge between the 4HB and Ig-like domains. The four helices in the IZUMO1 4HB domain (α1, α2, α3 and α4) vary from 14 to 30 residues in length. The helices are amphipathic in character with a solvent-exposed polar surface and hydrophobic residues packed in a core [16].

Generally, in immunolocalization studies, the samples were divided in capacitated or reacted (spontaneous or induced) spermatozoa and fresh spermatozoa as control group. Comparison between this groups shows the relocation of IZUMO1 during the AR. A few articles [40,42] compare cryopreserved and fresh spermatozoa (control). These studies indicate the effects of the cryopreservation, such as changes in localization and density of IZUMO1. 

There are slight differences in the localization of IZUMO1 between species. In mouse spermatozoa IZUMO1 is originally localized in the acrosomal membrane (inner and outer acrosomal membranes) [18]. While in bull spermatozoa it is detected along the border between the principal and EQ of the acrosomal region [40]. During the AR IZUMO1 gradually moves with the help of the actin cytoskeleton and testis-specific serine kinase (TSSK6) towards the EQ [43,44]. It has been observed in rodents that the beginning of IZUMO1 relocation does not depend on the beginning of the AR, however, later stages of relocation correlate positively with the status of the AR. The complete translocation in the EQ coincides with the completion of the AR [43]. 

IZUMO1 relocation in individual sperm populations during the spontaneous AR correlates with species-specific promiscuity behaviour, exhibiting faster relocation. Notably, in rodents, induction of the AR by calcium ionophore provides comparable or identical results to the spontaneous AR, whereas induction by progesterone completes relocation 20 min faster than in the spontaneous AR [43]. Therefore, it is suggested that IZUMO1 relocation is independent of external or internal calcium ion triggering the AR. While progesterone, a hormone secreted by cumulus oophorus cells responsible for the induction of the AR, is able to initiate IZUMO1 relocation. 

The location of IZUMO1 can be affected by several factors. One of them is the loss of sperm equatorial segment protein 1 (SPESP1), an acrosomal protein that localizes to the EQ after the AR. Its loss in both *Spesp1*^−/−^ and *Spesp1*^−/+^ mice results in an altered localization of IZUMO1, being distributed over a wider area and in an irregular manner. This event results in an inhibition of sperm–oocyte fusion, observed in both mouse and human sperm [45]. 

On the other hand, ablation of *Tssk6* causes the disappearance of actin polymerization in the sperm midpiece and postacrosomal region. Actin polymerization is necessary for the relocalization of IZUMO1; therefore, their alterations result in male infertility [44]. Another molecule that forms stable complexes with IZUMO1 is GLIPR1-like protein 1 (GLIPR1L1). GLIPR1L1 is required to perform optimal fertilization, as deletion of this protein leads to dysregulation of acrosomal exocytosis, failure of IZUMO1 relocalization and low in vitro fertilization rates [46]. This relationship between IZUMO1 and other fertilization proteins are summarized in Table 3.

**Table 2:** Articles studying IZUMO1 characterization and localization.

Ref.	Specie	Age	Sample	Main Findings
[16]	Human	n.d.	PS	- Crystal structures of human IZUMO1 and JUNO in unbound and bound conformations were found. - Mutational studies at the IZUMO1–JUNO interface revealed the structural determinants required for binding. - Biophysical characterization of IZUMO1 revealed a stable and monomeric protein with extensive mixed α –β secondary structural characteristics. - Human IZUMO1 does not have predictive properties of viral, intracellular or developmental fusogens.
[39]	Mouse Rat Hamster	Adult	CE	- Three novel proteins were identified (IZUMO 2, 3 and 4) showing the IZUMO domain. - IZUMO1, 2 and 3 are transmembrane proteins specifically expressed in the testis. IZUMO4 is a soluble protein expressed in the testis and other tissues. - Co-immunoprecipitation studies showed the presence of sperm proteins associated with IZUMO1, suggesting IZUMO1 forms a multiprotein membrane complex. - IZUMO1 might be involved in organizing or stabilizing a multiprotein complex essential for the membrane fusion.
[40]	Bovine ^a^ (*n* = 15)	>1 year	E EF	- Four patterns of IZUMO1 localization were identified: along the border between the principal and EQ of the acrosomal region (P1), the whole EQ (P2), the whole acrosomal region (P3) and absent (P4). - In epididymis and freshly ejaculated sperm with normal acrosomes, P1 was the predominant pattern. After the AR induction, an increase of sperm without the acrosome and P2 sperm was noted. - Bull IZUMO1 undergoes maturation-related changes during sperm transit through the epididymis and that it is translocated to the EQ of the acrosomal region during the AR. - Impaired fertilizing ability of bull cryopreserved spermatozoa with damaged acrosome is partially related to the aberrant translocation of IZUMO1.
[41]	Mouse ^b^	>12 weeks	CE VD	- A new *Izumo1* splicing variant was discovered (IZUMO1_v2) with a unique 52-amino-acid-long signal sequence transcribed from Exon 1b. - A small fraction of IZUMO1 is sufficient for triggering the sperm–oocyte fusion. - IZUMO1_v2 might function as a fail-safe in mouse for when splicing is disturbed.
[43]	Mouse ^c^	10–12 weeks	Distal regions of CE	- IZUMO1 is relocated from the acrosomal cap to the EQ and further over the whole sperm head during spontaneous AR. - The beginning and the progress of IZUMO1 relocation and tail TyrP were positively correlated with the level of promiscuity and the acrosome instability in promiscuous species. - In terms of time, there were no differences in IZUMO1 relocation when comparing the induced and spontaneous acrosome reacted sperm. IZUMO1 relocation displayed a different pattern and was initiated and completed earlier in the progesterone-induced group.
[47]	Wistar rat	8–10 weeks	E	- IZUMO1 is phosphorylated on residue S339 in the caput and corpus but not in caudal sperm cells. - IZUMO1 exhibited four phosphorylated residues when spermatozoa reached the cauda, which were absent from caput cells. - These phospho-regulations are likely to act as a scaffold for the association of adaptor proteins with IZUMO1 as these cells prepare for fertilization.
[48]	Mouse	8 weeks	WT sperm ΔCyt/ΔCyt sperm	- Truncated IZUMO1 showed identical location with WT IZUMO1 even after AR. - Mice without the cytoplasmic tail of IZUMO1 showed normal fertility but decreased the amount of protein, indicating that this region is important for the expression level of IZUMO1.
[49]	Datong yak (*n* = 16)	6, 18, 30 and 72 months	Multitissues ^d^	- *Izumo1* expression might be higher during the peak breeding ages (6 to 7 years) of the yak and play a potential role in spermatogenesis, fertility and testicular development. - The secondary and tertiary protein structure prediction revealed the presence of a signal peptide, an IZUMO domain, an immunoglobulin like domain and a transmembrane region.
[50]	Sheep ^e^ (*n* = 760)	3 years	DNA	- *Izumo1* has nine exons. - *Izumo1* and *Juno* are very conservative among *Bovidae*. - The expression of IZUMO1 was higher in the testis than in all other tissues. - The signal peptide region at the N-terminal and the transmembrane region at the C- terminal of IZUMO1 had strong hydrophobic regions.
[42]	Piétrain boar (*n* = 12)	Different sexually mature	EF	- Cryopreservation induces changes in IZUMO1 localization. - The relative content of IZUMO1 was not altered after thawing. - IZUMO1 relocates during cryopreservation, which could contribute to a reduced fertilising capacity of frozen-thawed boar sperm.
[51]	Ram	n.d.	T E EF	- Frozen-thawed samples had lower density and expression than the EF samples. - The expression of IZUMO1 was seen as an increased band formation from the equatorial region through the acrosome, after in vitro capacitation. - After the AR, the band was only present in the equatorial region.
[52]	Mouse ^f^	Retired male breeders	CE	- IZUMO was widespread and was found in intermediate-density fractions. - After the AR, IZUMO was distributed along the sperm head, reaching the post- and para-acrosomal areas. - The diversity off immunofluorescence patterns were due to the AR and were not directly related to the capacitation process.
[53]	Bovine	n.d.	Multitissues ^g^	- The C-terminal sequence of bovine IZUMO1 is quite different among other species, suggesting that it may have a distinct function. - Bovine IZUMO1 is expressed on the surface of sperm as a homodimeric complex. - Complex formation of IZUMO1 may be necessary for retaining the protein conformation, which in turn, might be contributing to sperm–oocyte fusion in some way.
[54]	Sheep (*n* = 5) Cashmere goat (*n* = 6)	12 months	T	- Alignment of IZUMO1 protein sequences among 15 mammalian species displayed several highly conserved regions. - IZUMO1 phosphorylation is not essential for its functionality. - IZUMO1 isoforms might have biological functions during spermatogenesis or spermiogenesis other than sperm–oocyte fusion in sheep and goats.
[55]	Boar ^h^	n.d.	Sperm Multitissues ^i^	- IZUMO1 is expressed on the surface of sperm as a homodimeric complex. - IZUMO1 is localized in EQ as well as on the inner acrosomal membrane. - C-terminal sequence of porcine IZUMO1 is quite different from other species, suggesting that it may have a distinct function.
[56]	Mouse	>8 weeks	CE	- IZUMO1_PFF_ interacts with fertilization inhibitory antibodies. - IZUMO1_PFF_ still maintains its binding ability on the oocyte surface of *Cd9*^−/−^ oocytes surface. - IZUMO1_PFF_ has an N-terminal unfolded structure and a C-terminal ellipsoidal helix dimer. The formation of a helical dimer at the N-terminal region of IZUMO1 is required for its function.
[57]	Mouse	n.d.	Sperm ^j^	- Dimeric IZUMO1 was already formed at the acrosomal cap region before the AR and it is redistributed to the EQ after the AR. - They categorized the dimer translocation into two types: Type 1, the near-simultaneous appearance of bimolecular fluorescence complementation signals with IZUMO1-mCherry; and Type 2, the delayed formation of dimer in the EQ.
[58]	Cashmere goat	n.d.	EF	- IZUMO1 was localised in the EQ of the sperm head surface.
[59]	Mouse ^k^	>8 weeks	Sperm from transgenic N204Q-IZUMO	- N204Q-IZUMO was located as WT IZUMO was. - Glycosylation is not essential for the function of IZUMO but has a role in protecting it from fragmentation in cauda epididymis.
[60]	Mouse	9–16 weeks	Sperm	- Human JUNO can interact with mouse IZUMO1. - Trp62 of JUNO participates in the interaction with IZUMO1.
[61]	Human (*n* = 20)	n.d.	Sperm from healthy men	- IZUMO1 was not affected during the vitrification process.

^a^ Japanese Black cattle; ^b^ B6D2F1 and ICR mouse; ^c^ BALB/c, *A. sylvaticus* and *A. microps*; ^d^ Testis, liver, kidney, lung, heart, spleen and intramuscular fat tissues; ^e^ Small Tail Han sheep (*n* = 360), Sunite sheep (*n* = 100), Tan sheep (*n* = 80), Suffolk sheep (*n* = 39), Dorper sheep (*n* = 30) and Prairie Tibetan sheep (*n* = 131); ^f^ CD1; ^g^ testis, liver, kidney, lung, heart, spleen, uterus and oviduct; ^h^ NIBS miniature pig; ^i^ brain, heart, lung, liver, kidney, uterus, oocyte and testis; ^j^ Sperm from *Izumo1* knockout, IZUMO1-His transgenic, IZUMO1-mCherry transgenic and Acro-GFP mice; ^k^ BDF1. Abbreviations: AR, acrosome reaction; CE, cauda epididymal sperm; E, epididymal; EF, ejaculated fresh sperm; EQ, equatorial segment; GFP, green fluorescent protein; IZUMO1_PFF_, IZUMO1 putative functional fragment; n.d., non-data; PS, protein sequence; T testicles; VD, vas deferens sperm; WT, wild type.

The effect of cryopreservation on IZUMO1 location has been observed in bull spermatozoa [40]. Cryopreservation generates severe damage to the acrosome, resulting in aberrant translocation of IZUMO1. After induction of the AR, a higher loss of acrosomes is observed in cryopreserved spermatozoa compared to freshly ejaculated samples. Consequently, the percentage of cryopreserved spermatozoa with normal or abnormal acrosome and a normal IZUMO1 distribution pattern decreases about a 9 ± 5%. Although the detailed mechanism for IZUMO1 translocation in cryopreserved sperm is still unclear, Fukuda et al. suggest that cryopreserved sperm undergo changes, such as the capacitation process induced by the freezing and thawing process and by exposure to components such as egg yolk [40].

#### 3.5.2. Role of IZUMO1 in Mammalian Fertilization

The role of IZUMO1 in mammalian fertilization has been studied in several articles (*n* = 11). As it is shown in Table 4 most of the studies have been conducted on epididymal spermatozoa or vas deference from mice with at least eight-weeks old. In addition, binding, fusion and adhesion assays were employed. Due to JUNO-IZUMO1 receptor is located in the oocyte surface, in these assays, mature oocytes from humans or mice with or without ZP were used. Nevertheless, the use of ZP-intact oocytes for gamete fusion in vitro studies is not suitable because the presence of the ZP difficult the control of sperm penetration and fusion timing [75]. To study IZUMO1, capacitated and reacted spermatozoa or transfected cells expressing IZUMO1 were incubated with oocytes (details in Table 4).

The molecular mechanism of recognition between IZUMO1 and JUNO was recently discovered, in which IZUMO1 interacts with JUNO through its N-terminal domain [56]. The crystal structure indicates that the residues of the three IZUMO1 regions (4HB, hinge and Ig-like) contact JUNO 20-228 through extensive van der Waals, hydrophobic and aromatic interactions. There are also two intermolecular salt bridges and eight hydrogen bonding interactions. However, all these interactions are weak [16]. On the other hand, Inoue et al. described two types of dimer configuration in IZUMO1: open and closed. The open dimer could increase JUNO affinity to increase the fertilization rate. While the closed dimer directly participates in membrane fusion after binding to JUNO [57]. 

First, JUNO binds to monomeric IZUMO1, gradually accumulating at the sperm contact site to induce its dimerization, which is followed by a tight junction phase in which IZUMO1 folds the entire structure to the sperm membrane side through a thiol-disulphide exchange reaction. Following this, IZUMO1 stops binding to JUNO to bind to a second putative oocyte receptor. 

Thus, the role of JUNO is to rearrange IZUMO1 so that it can generate the necessary strength to collapse the repulsion between the juxtaposing membranes through an unidentified receptor on the oocyte. However, this is not sufficient to fuse both membranes, requiring other proteins, such as CD9 in the oocyte and SPACA6 in the sperm [12,13,76,77]. CD9, IZUMO1 and JUNO are involved in gamete recognition and membrane adhesion, but do not induce membrane fusion [15]. Interestingly, CD9 has been shown to regulate the interaction between JUNO and IZUMO1 in wild-type mice [78].

Recently, the structure of the human JUNO/IZUMO1 complex has been reported and critical amino acids have been identified during the two molecules interaction [16,17]. According to structural data, mutations in W148, H157 and R160 of IZUMO1 and W52 and L81 of JUNO are essential for sperm and oocyte plasma membrane recognition [17]. 

After fusion, the sperm membrane comprises a continuous single membrane plane with a complicated invaginated structure. However, sperm–oocyte fusion is not completely achieved at this point, as electron microscopy images have showed, the “internalization” of the invaginated inner acrosomal membrane occurs later in the fertilization process [79].

**Table 4:** Articles studying IZUMO1 function.

Ref.	Specie	Age (Weeks)	Sample	Main Findings
[8]	Human Mouse Boar	n.d.	PS	- IZUMO1 and JUNO interaction plays an important role in recognition and fusion in different species.
[18]	Mouse	n.d.	E	- IZUMO1 translocation and the sperm–oocyte fusion event were imaged live. - IZUMO1 translocation happened from the acrosomal membrane to the plasma membrane.
[33]	Human	n.d.	Sperm ^a^	- A possible correlation between *IZUMO* polymorphism and ICSI outcome was found. Their results did not confirm this hypothesis as there is a no favourable *IZUMO* polymorphism for ICSI outcome. - Infertility is not due to a defective IZUMO. Mutations in other parts of the gene cannot be discarded.
[34]	Mouse	8	CE	- In sperm, IZUMO1 is monomeric while it is dimeric during the adhesion to the oocyte. - JUNO associates with monomeric IZUMO1, which is then quickly removed as a tight adhesion of the two cells is established. - A global structural rearrangement of IZUMO1 occurs during JUNO recognition and this may provide enough strength to overcome the repulsion between the juxtaposing membranes.
[78]	Mouse	8–10	CE VD	- IZUMO1 and JUNO are interchangeable between mice and humans. - The high robustness of the IZUMO1-induced cell-oocyte adhesion takes place within the first minutes of contact with JUNO. - The role of IZUMO1 should therefore be to generate a direct robust cell adhesion that would trigger a molecular organization suitable for the fusion, in the contact area of the sperm and the oocyte. - They suggested that by inducing adhesion to JUNO, IZUMO1 is accumulated at the fusion site and triggers the recruitment of CD9, both thereby conveying their own cis partners to build the gamete fusion machinery.
[80]	Mouse ^b^	12–24	CE	- Anti-IZUMO1 gave no immunostaining signal on live sperm. The protein was recognised on the sperm head following the AR. - It is possible that the spread of IZUMO1 may occur immediately after fusion between the outer acrosomal and plasma membranes of sperm, even if some of the acrosomal proteins remain in the matrix. - They could not observe penetration of the above two acrosome-reacted sperm into the ZP.
[81]	Mouse	>8	Sperm ^c^	- *Izumo1*-disrupted sperm cannot fertilize the oocyte.
[82]	Mouse ^b^	12–14	CE	- Cleavage of SPACA1 regulates IZUMO1 translocation to the equatorial segment. - During IZUMO1 translocation, IZUMO1 epitope were externalized from the acrosomal lumen before acrosomal vesiculation and was phosphorylated.
[83]	Human	n.d.	PS	- They predicted protein–protein interactions, such as IZUMO1-CD9 and ADAM2-IZUMO1 that may play an important role in sperm–oocyte interaction. - ADAM2 may mediate interaction between CD9 and IZUMO1.
[84]	Boar ^d^	n.d.	E	- The proportion of sperm that were immunopositive for the anti-IZUMO antibody was higher after they were passing or had passed through the ZP. - Addition of anti-IZUMO antibody to the fertilization medium inhibited the sperm penetration into ZP-free oocytes.
[85]	Mouse	>10–12	CE VD	- Fecundity was positively correlated with IZUMO1 protein levels.

^a^ Normozoospermic infertile patients, infertile patients requiring ICSI, normozoospermic, fertile control and fertile men from general population; ^b^ ICR mouse; ^c^ *Izumo1*^−/−^ sperm collected from PVS wild type sperm collected from PVS of *Cd9*^−/−^ oocyte; ^d^ Landrace boar. Abbreviations: ADAM2, disintegrin and metalloproteinase domain-containing protein 2; AR, acrosome reaction; CE, cauda epididymal sperm; E, epididymal sperm; ICSI, intracytoplasmic sperm injection; n.d., non-data; PS, protein sequence; PVS, perivitelline space; SPACA1, Sperm acrosome membrane-associated protein 1; VD, vas deferens sperm; ZP, zona pellucida.

## 4. Future Directions

The characterization and description of IZUMO1 and TMEM95—sperm proteins required for mammalian gamete fusion—provides relevant insight into the gamete fusion process. However, future studies are needed to increase the knowledge of sperm receptors and to understand their role during fertilization. Even though coimmunoprecipitation analyses suggest an interaction between TMEM95 and IZUMO1, no evidence has been achieved by AVEXIS [22]. 

Therefore, other types of assays should be performed to verify whether they interact via their transmembrane domain. Nevertheless, the expression of these proteins is not sufficient for sperm–oocyte fusion. Thus, there must be other molecules involved in the process. Recently, Matsumura et al. published that a small population of the acrosome-reacted *Izumo1*-KO mouse spermatozoa can still adhere to the oolemma, suggesting that additional mechanisms of sperm–oolemma adhesion exist on top of the IZUMO1-JUNO interaction [86]. 

Currently, the exact topological location of IZUMO1 is unknown, differing among species and physiological conditions. In addition, it is not known whether the distribution of IZUMO1 is homogeneous or heterogeneous. In this context, high-resolution electron microscopy (FE-SEM) and transmission electron microscopy (TEM) could be used to elucidate the localization of IZUMO1 during capacitation, fertilization and AR. FE-SEM offers a valuable method to study sperm proteins by detecting the presence of sperm proteins qualitatively and quantitatively using colloidal gold [87,88].

Overall, understanding the molecular and cellular basis of gamete fusion through further research will enable researchers to propose new treatments for infertility [89] and to develop new contraceptive strategies. Research has proposed the use of sperm-specific proteins such as IZUMO1 to create new non-steroidal and reversible contraceptive methods. The research reported that these contraceptive vaccines can be used in both males and females [90,91,92,93,94,95,96].

## Figures and Tables

**Table 1:** Articles studying TMEM95 in different species.

Ref.	Specie	Study Groups	Immuno- Localization	Main Findings
[20]	Mouse	Multitissue ^a^	Confocal laser scanning microscopy	- Transgenic mice that expressed TMEM95 rescued the sterility of *Tmem95* knockout males. - The coexpression of SOF1, TMEM95 and SPACA6 in IZUMO1-expressing cultured cells did not enhance their ability to attach the oocyte membrane or allow them to fuse with oocytes. - SOF1, TMEM95 and SPACA6 may function co-operatively with IZUMO1 and/or unknown fusogens in sperm−oocyte fusion.
[22]	Mouse	Fresh sperm (*n* = 20) Cryopreserved sperm (*n* = 20)	Epifluorescence inverted microscopy	- TMEM95 relocalizes to the equatorial region after the AR. - Sperm lacking TMEM95 were morphologically normal exhibited normal motility and could penetrate the zona pellucida and bind to the oolemma. - TMEM95-deficient sperm cannot fuse with oocytes. - TMEM95 does not interact with JUNO or IZUMO1. - TMEM95 is essential for mammalian fertilization.
[23]	Bovine	Cryopreserved sperm WT (*n* = 10) Hz (*n* = 10) Mt (*n* = 13)	Confocal laser scanning microscopy	- Causal nonsense mutation (rs378652941, c.483C > A, p.Cys161X) in *Tmem95*. - TMEM95 in fertile animals is located on the sperm surface, whereas it is absent in the spermatozoa of subfertile animals. - Integrity of TMEM95 is required for a successful fertilisation.
[24]	Bovine	WT (*n* = 2) Hz (*n* = 3) Mt (*n* = 5)	Upright microscopy	- Sperm from Mt males had lower in vitro fertility than sperm from WT or Hz bulls. - Early embryo division was abnormal in the Mt group. - TMEM95 is lost after the AR, and thus it might be involved in events that lead to sperm–oocyte interaction.
[31]	Bovine	Multitissue ^b^ (*n* = 3)	n.a.	- Identified two transcripts of *Tmem95*: TMEM95-SV1 and TMEM95-SV2. - TMEM95-SV1 has a leucine-rich repeat C- terminal domain and a Pfam: IZUMO domain. - The two transcripts were exclusively expressed in the testes and brain in male foetus cattle.
[32]	Boar ^c^	Testis (*n* = 289)	n.a.	- NC_010454.3: g.341T > C Synonymous mutation (A47A). - Significant relationship between A47A polymorphism and testis weight. - *Tmem95* could be a gene candidate associated with reproductive characteristics.
[35]	Buffalo	Herford cattle genome sequence	n.a.	- Non-synonymous SNPs could affect the protein function. - *Tmem95* in cattle and buffalo must have evolved with different functions but plays a role in male fertility as in other mammalians.
[36]	Bovine ^d^	DNA (*n* = 765)	n.a.	- Newly frameshift insertion/deletion (indel) mutation (NC_037346.1: g.27056998_27057000delCT) in *Tmem95* in 11 cattle breeds. - This study provides the evidence that in Chinese cattle breeds the mutation c.483C > A cannot be used as a genetic marker in molecular breeding.

^a^ Brain, thymus, lung, heart, liver, spleen, kidney, testis, epididymis (caput, corpus and cauda regions), seminal vesicle, prostate (mixture of dorsal, lateral and ventral regions), anterior prostate, ovary and uterus; ^b^ Heart, liver, spleen, lung, kidney, muscle, testis and brain; ^c^ Landrace and Large White piglets; ^d^ Red Steppe cattle (*n* = 135), Qinchuan cattle (*n* = 60), Nanyang cattle (*n* = 60), Jinnan cattle (*n* = 60), Luxi cattle (*n* = 30), Xia’nan cattle (*n* = 60), Jiaxian Red cattle (*n* = 60), Pi’nan cattle (*n* = 60), Jinjiang cattle (*n* = 60), De’nan cattle (*n* = 30), Yunling cattle (*n* = 60), Zaosheng cattle (*n* = 30) and Bohai Black cattle (*n* = 60). Abbreviations: AR, acrosome reaction; Hz, heterozygote; Mt, mutated; n.a. non applicable; Pfam, database of protein families and domains; SOF1, Sperm-egg fusion protein LLCFC1; SPACA6, Sperm acrosome membrane-associated protein 6; TMEM95, transmembrane protein 95; and WT, wild type.

**Table 3:** Articles studying the relationship between IZUMO1 and other proteins.

Ref.	Specie	Protein	Main Findings
[21]	Mouse	FIMP	- IZUMO1 translocation was normal in *Fimp* knockout spermatozoa. - IZUMO1 was present and located properly in the transmembrane-deleted mice, as well as wild-type mice. - Although IZUMO1 was still intact, *Fimp* knockout spermatozoa lacked the ability to fuse with oocytes. - IZUMO1-expressing cells directly bind to the oocyte surface.
[38]	Mouse	SPACA6	- Relocation of IZUMO1 is not affected by the lack of SPACA6. - A model was proposed where IZUMO1 and SPACA6 would be part of an essential molecular complex for gamete fusion. Their concomitant presence would be required for the recruitment of another essential molecules for the sperm–oocyte fusion.
[44]	Mouse	TSSK6	- In the absence of TSSK6, IZUMO fails to relocate after the AR.
[46]	Mouse	GLIPR1L1	- GLIPR1L1 is required for IZUMO1 redistribution after AR.
[62]	Mouse	ADAM3	- ADAM3 and IZUMO1 were found exclusively in sperm heads. - Proteins that contain a transmembrane domain, e.g., IZUMO1 and CD46, were distributed in detergent-depleted and detergent-enriched phase.
[63]	Mouse	ACE3	- ACE3 interacts with IZUMO1. - The IZUMO1 location in *Ace*3^−/−^ sperm spreads a wider area.
[64]	Mouse	INPP5B	- IZUMO1 appears normal in *Inpp5b*-null sperm.
[65]	Mouse	CAPZA3	- CAPZA3 movement precedes IZUMO1 relocation.
[66]	Mouse	TMEM190	- TMEM190 is co-localized with IZUMO1 in mouse sperm before and after the AR. - TMEM190 immunoprecipitation did not include IZUMO1.
[67]	Mouse	TPST2	- The location of sulphated tyrosines on sperm is similar to IZUMO1 location. - Very little or none of IZUMO1 is sulphated. - IZUMO1 expression and location are normal in Tpst2-null sperm.
[68]	Mouse	LatA	- 25 μM LatA blocked actin polymerization in capacitated sperm head. This results in a marked decrease in the number of sperm with relocated IZUMO1 during the A23187-induced AR. - Treated sperm also exhibited a reduced cumulus layer and a lower zona pellucida penetration and fertilizing capacity. - LatA-treated sperm at the perivitelline space of oocytes did not show impaired IZUMO1 relocation.
[69]	Mouse	EQTN	- IZUMO1 and CD9 are present in *Eqtn*^−/−^ sperm that reached the perivitelline space. - Immunostaining of IZUMO1 was aberrant in the early to middle stage but normal in the late stage of the AR in *Eqtn*^−/−^ sperm. - *Eqtn/Spesp1*^−/−^ sperm showed an abnormal IZUMO1 immunostaining pattern in the head after the AR, but they were normal when examined by electron microscopy. - EQTN and IZUMO1 may play different roles in the sperm–oocyte adhesion to fusion process.
[70]	Human	Dpy19I2	- IZUMO family proteins (IZUMO 1–4) are downregulated, with little or no expression in Dpy19l2-deficient globozoospermia.
[71]	Mouse	DCST1/2 SPACA6	- Loss of SPACA6 is recovered by IZUMO1 complementation (*IZUMO1*^−/−^*IZUMO1*-transgenic). - IZUMO1 and SPACA6 might to be cooperative factors. - SPACA6 stability is differently regulated by DCST1/2 and IZUMO1.
[72]	Mouse	PtdSer	- IZUMO1 and PtdSer may be present on the sperm surface at the same time for subsequent interactions with the oocyte. - In sperm, IZUMO1 and PtdSer could function cooperatively mediating the sperm–oocyte binding and fusion.
[73]	Mouse	CD9	- The lack of CD9 likely affects the binding phase of IZUMO1 resulting in a lower number of binding cells. - The IZUMO1–JUNO interaction is not impaired by *Cd9* disruption; therefore, IZUMO1–JUNO and CD9 may be independent pathways for triggering the sperm–oocyte fusion.
[74]	Bovine	OMC32	- IZUMO1 is located over the equatorial segment. - After the AR, IZUMO1 remains associated to the particulate fraction. - IZUMO1 relocates to the equatorial segment during the lysophosphatidylcholine-induced AR.

Abbreviations: ACE3, angiotensin-converting enzyme-like protein Ace3; ADAM3, a disintegrin and metallopeptidase domain 3; AR acrosome reaction; CAPZA3, f-actin-capping protein subunit alpha-3; DCST1/2, E3 ubiquitin-protein ligase DCST1/2; Dpy19I2, probable C-mannosyltransferase DPY19L2; EQTN, equatorin; FIMP, Fertilization-influencing membrane protein; GLIPR1L1, GLIPR1-like protein 1; INPP5B, Type II inositol 1,4,5-trisphosphate 5-phosphatase; LatA, latrunculin A; OMC32, 32-kDa acrosomal matrix protein; PtdSer, phosphatidylserine; SPACA6, Sperm acrosome membrane-associated protein 6; SPESP1, sperm equatorial segment protein 1; TMEM190, transmembrane protein 190; TPST2, Protein-tyrosine sulfotransferase 2; TSSK6, testis-specific serine kinase.

## Data Availability

Not applicable.

## Supplementary Materials

The following supporting information can be downloaded at: https://www.mdpi.com/article/10.3390/ijms23073929/s1.
