# Supplementary material for: The Role of Sperm Proteins IZUMO1 and TMEM95 in Mammalian Fertilization: A Systematic Review
Source: Int J Mol Sci. 2022 Apr 1;23(7):3929. doi: 10.3390/ijms23073929 (PMC8999778; doi:10.3390/ijms23073929)
Supplement: Supplementary file 1 [file ijms-23-03929-s001.zip › ijms-1647856-supplementary.pdf]

Table S1. Author/s, year, and tittle of the 8 articles analysed in this study that studied TMEM95.

Reference	Tittle
Fernandez-Fuertes et al. 2017	Subfertility in bulls carrying a nonsense mutation in transmembrane protein 95 is due to failure to interact with the oocyte vestments
Lamas-Toranzo et al. 2020	TMEM95 is a sperm membrane protein essential for mammalian fertilization
Liu et al. 2017	A novel synonymous SNP (A47A) of the TMEM95 gene is significantly associated with the reproductive traits related to testis in male piglets
Noda et al. 2020	Sperm proteins SOF1, TMEM95, and SPACA6 are required for sperm-oocyte fusion in mice
Pausch et al. 2014	A nonsense mutation in TMEM95 encoding a nondescript transmembrane protein causes idiopathic male subfertility in cattle
Shireesha et al. 2021	Bioinformatic characterization of the Transmembrane protein95 gene (TMEM95) in Murrah buffalo (Bubalus bribalis)
Zhang et al. 2016	Identification of novel alternative splicing transcript and expression analysis of bovine TMEM95 gene
Zhang et al. 2019	Detection of Bovine TMEM95 p.Cys161X Mutation in 13 Chinese Indigenous Cattle Breeds

Table S2. Author/s, year, and tittle of the 57 articles analysed in this study that studied IZUMO1.

Reference	Tittle
An et al. 2009	In vitro and in vivo studies evaluating recombinant plasmid pCXN2-mIzumo as a potential immunocontraceptive antigen
Aydin et al. 2016	Molecular architecture of the human sperm IZUMO1 and egg JUNO fertilization complex
Baker et al. 2012	Analysis of phosphopeptide changes as spermatozoa acquire functional competence in the epididymis demonstrates changes in the post-translational modification of Izumo1
Barbaux et al. 2020	Sperm SPACA6 protein is required for mammalian Sperm-Egg Adhesion/Fusion
Bianchi and Wright 2015	Cross-species fertilization: the hamster egg receptor, Juno, binds the human sperm ligand, Izumo1
Chalbi et al. 2014	Binding of sperm protein Izumo1 and its egg receptor Juno drives Cd9 accumulation in the intercellular contact area prior to fusion during mammalian fertilization
Clark and Naz 2013	Presence and incidence of izumo antibodies in sera of immunoinfertile women and men
Ellerman et al. 2009	Izumo is part of a multiprotein family whose members form large complexes on mammalian sperm
Fard and Shamsir 2013	Construction and Analysis of the Cell Surface's Protein Network for Human Sperm-Egg Interaction
Fujihara et al. 2020	Spermatozoa lacking Fertilization Influencing Membrane Protein (FIMP) fail to fuse with oocytes in mice
Fukuda et al. 2016	Changes of IZUMO1 in bull spermatozoa during the maturation, acrosome reaction, and cryopreservation
Gaikwad et al. 2019	GLIPR1L1 is an IZUMO-binding protein required for optimal fertilization in the mouse
Granados-Gonzalez et al. 2008	Preliminary study on the role of the human IZUMO gene in oocyte-spermatozoa fusion failure
Gundogan and Aktas 2021	Immunolocalization of Fertilin β, IZUMO1, and P34H in Ram Spermatozoa
Guo et al. 2019	Proteomic Analysis of Dpy19l2-Deficient Human Globozoospermia Reveals Multiple Molecular Defects
Hu et al. 2021	Expression, structure and function analysis of the sperm-oocyte fusion genes Juno and Izumo1 in sheep (Ovis aries)
Inoue et al. 2010	Identification and disruption of sperm-specific angiotensin converting enzyme-3 (ACE3) in mouse
Inoue et al. 2011	Acrosome-reacted mouse spermatozoa recovered from the perivitelline space can fertilize other eggs
Inoue et al. 2013	Molecular dissection of IZUMO1, a sperm protein essential for sperm-egg fusion
Inoue et al. 2015	Oocyte-triggered dimerization of sperm IZUMO1 promotes sperm-egg fusion in mice
Inoue, Hagihara and Wada 2021	Evolutionarily conserved sperm factors, DCST1 and DCST2, are required for gamete fusion
Inoue, Ikawa and Okabe 2008	Putative sperm fusion protein IZUMO and the role of N-glycosylation
Inoue, Saito, and Wada 2020	Unveiling a novel function of CD9 in surface compartmentalization of oocytes
Inoue and Wada 2018	Monitoring dimeric status of IZUMO1 during the acrosome reaction in living spermatozoon
Ito et al 2018	Deletion of Eqtn in mice reduces male fertility and sperm-egg adhesion
Kalgar et al. 2019	Expression Analysis of IZUMO1 Gene during Testicular Development of Datong Yak (Bos Grunniens)

Table S2. *Cont.*

Reference	Tittle
Kato et al. 2016	Structural and functional insights into IZUMO1 recognition by JUNO in mammalian fertilization
Kim et al. 2013	Molecular cloning, characterization of porcine IZUMO1, an IgSF family member
Kim 2015	Molecular cloning and characterization of Izumo1 gene from bovine testis
Kumar et al. 2021	Unraveling Subcellular and Ultrastructural Changes During Vitrification of Human Spermatozoa: Effect of a Mitochondria-Targeted Antioxidant and a Permeable Cryoprotectant
Llavanera et al. 2019	GSTM3, but not IZUMO1, is a cryotolerance marker of boar sperm
Marcello and Evans 2010	Multivariate analysis of male reproductive function in Inpp5b-/- mice reveals heterogeneity in defects in fertility, sperm-egg membrane interaction and proteolytic cleavage of sperm ADAMs
Marcello et al. 2011	Lack of tyrosylprotein sulfotransferase-2 activity results in altered sperm-egg interactions and loss of ADAM3 and ADAM6 in epididymal sperm
Miranda et al. 2009	Localization of low-density detergent-resistant membrane proteins in intact and acrosome-reacted mouse sperm
Mortazavi et al. 2021	Evaluation of multi-epitope recombinant protein as a candidate for a contraceptive vaccine
Nagdas et al. 2016	Identification of bovine sperm acrosomal proteins that interact with a 32-kDa acrosomal matrix protein
Naz 2008	Immunocontraceptive effect of izumo and enhancement by combination vaccination
Naz 2014	Vaccine for human contraception targeting sperm Izumo protein and YLP12 dodecamer peptide
Nishimura et al. 2011	Characterization of mouse sperm TMEM190, a small transmembrane protein with the trefoil domain: evidence for co-localization with IZUMO1 and complex formation with other sperm proteins
Rival et al. 2019	Phosphatidylserine on viable sperm and phagocytic machinery in oocytes regulate mammalian fertilization
Saito, Wada and Inoue 2019	Alternative splicing of the Izumo1 gene ensures triggering gamete fusion in mice
Saito, Wada, and Inoue 2019	Sperm IZUMO1-Dependent Gamete Fusion Influences Male Fertility in Mice
Satouh et al. 2012	Visualization of the moment of mouse sperm-egg fusion and dynamic localization of IZUMO1
Sebkova et al. 2014	Progress of sperm IZUMO1 relocation during spontaneous acrosome reaction
Sosnik, Buffone and Visconti 2010	Analysis of CAPZA3 localization reveals temporally discrete events during the acrosome reaction
Sosnik et al. 2009	Tssk6 is required for Izumo relocalization and gamete fusion in the mouse
Tanihara et al. 2014	Roles of the zona pellucida and functional exposure of the sperm-egg fusion factor 'IZUMO' during in vitro fertilization in pigs
Thérien and Manjunath 2003	Effects of latrunculin A on the relocation of sperm IZUMO1 during gamete interaction in mouse
Wang et al. 2008	Investigation of recombinant mouse sperm protein izumo as a potential immunocontraceptive antigen
Wang et al. 2009	Immunocontraceptive potential of the Ig-like domain of Izumo
Xing et al. 2011	Molecular cloning and characterization of Izumo1 gene from sheep and cashmere goat reveal alternative splicing
Xue et al. 2016	Vaccination with an Epitope Peptide of IZUMO1 to Induce Contraception in Female Mice
Yamaguchi et al. 2006	Aberrant Distribution of ADAM3 in Sperm from Both Angiotensin-Converting Enzyme (Ace)- and Calmegin (Clgn)-Deficient Mice
Yamashita et al. 2007	Acrosome reaction of mouse epididymal sperm on oocyte zona pellucida
Yamatoya et al. 2020	Cleavage of SPACA1 regulates assembly of sperm-egg membrane fusion machinery in mature spermatozoa†
Young et al 2016	CRISPR/Cas9-mediated mutation revealed cytoplasmic tail is dispensable for IZUMO1 function and male fertility
Zhi-da et al. 2010	Prokaryotic Expression, Ascitic Polyclonal Antibody Preparation and Identification of Cashmere Goat Izumo1

Table S3. Author/s, year, and tittle of the 19 reviews analysed in this study that reviewed IZUMO1.

Reference	Tittle
Bianchi and Wright 2016	Sperm Meets Egg: The Genetics of Mammalian Fertilization
Cuasnicú et al. 2016	Acrosome Reaction as a Preparation for Gamete Fusion
Evans 2011	Sperm-egg interaction
Harada et al. 2013	Critical role of exosomes in sperm-egg fusion and virus-induced cell-cell fusion
Inoue 2016	Novel insights into the molecular mechanism of sperm-egg fusion via IZUMO1
Inoue, Ikawa, and Okabe 2011	The mechanism of sperm-egg interaction and the involvement of IZUMO1 in fusion
Ito and Toshimori 2016	Acrosome markers of human sperm
Jiménez-Movilla, Hamze, and Romar 2021	Oolemma Receptors in Mammalian Molecular Fertilization: Function and New Methods of Study
Klinovska, Sebkova and Dvorakova-Hortova 2014	Sperm-egg fusion: a molecular enigma of mammalian reproduction
Liu 2015	Capacitation-Associated Glycocomponents of Mammalian Sperm
Mou and Xie 2017	Male infertility-related molecules involved in sperm-oocyte fusion
Muro and Okabe 2013	Mechanisms of Fertilization-A View From the Study of Gene-Manipulated Mice
Okabe 2013	The cell biology of mammalian fertilization
Sabetian and Shamsir 2017	Deficiency in Sperm–Egg Protein Interaction as a Major Cause of Fertilization Failure
Satouh and Ikawa 2018	New Insights into the Molecular Events of Mammalian Fertilization
Sun et al. 2019	Effects of sperm proteins on fertilization in the female reproductive tract
Toshimori 2011	Dynamics of the mammalian sperm membrane modification leading to fertilization: a cytological study
Yeste et al. 2017	Oocyte Activation and Fertilisation: Crucial Contributors from the Sperm and Oocyte
Young, Aitken and Baker 2015	Phosphorylation of Izumo1 and Its Role in Male Infertility

Table S4. Author/s, year, and tittle of the 12 other type of publication — letter, chapter, comment, correspondence— analysed in this study that studied IZUMO1.

Reference	Tittle
Bianchi and Wright 2014	Izumo meets Juno: preventing polyspermy in fertilization
Gerhardt 2016	IZUMO1-JUNO Union Promotes Fertilization
Gupta 2014	Unraveling the intricacies of mammalian fertilization
Hayasaka et al. 2007	Positive expression of the immunoglobulin superfamily protein IZUMO on human sperm of severely infertile male patients
Inoue et al. 2005	The immunoglobulin superfamily protein Izumo is required for sperm to fuse with eggs
Inoue and Okave 2010	Gamete fusion and sperm protein IZUMO1
Melcher 2016	Structural biology: When sperm meets egg
Miyado et al. 2018	Regulation of Sperm-Egg Fusion at the Plasma Membrane
Nishimura et al. 2016	The structure of sperm Izumo1 reveals unexpected similarities with Plasmodium invasion proteins
Ohto et al. 2016	Structure of IZUMO1-JUNO reveals sperm-oocyte recognition during mammalian fertilization
Schultz and William 2005	Developmental biology: sperm-egg fusion unscrambled
Wassarman 2014	Reproductive biology: Sperm protein finds its mate
